# Progress in Bacteriology

**Published:** 1901-08-17

**Authors:** 


### Progress in Bacteriology.

**Scarlet Fever.**—The evidence supporting a streptococcal origin of this disease is increasing year by year. Boginsky and Sommerfeld[1] summarise their results as follows:—Streptococci are present in all cases of faucial angina; in 42 fatal cases of scarlet fever streptococci were present in the blood and organs in every instance; the streptococcus of scarlet fever cannot be differentiated from other streptococci—the organism is virulent. Gordon,[2] on the other hand, maintains that the scarlatinal streptococcus has distinctive features, inasmuch as it shows marked conglomerative action in cultures, and when injected into mice may take on a bacillary form. It also differs from the streptococcus pyogenes in clotting milk, which the latter does not do. It is found in the blood and organs of fatal cases of scarlatina, and out of 10 such cases investigated, this, the streptococcus conglomeratus, was identified in five, whilst in the remainder an organism probably identical with it was present.

**Cerebro-spinal Meningitis.**—Hunter and Nuthall[3] have investigated 10 cases of this disease. In nine the fluid drawn off by lumbar puncture was found to contain micro-organisms, diplococci of two varieties. The one variety of diplococcus was not stained by Gram, formed fine transparent colonies on agar, grew poorly in broth and on potato, and on blood serum. The second variety stained easily with Gram, gave rise to luxuriant growth on agar, blood, and potato, and in bouillon. Both varieties had capsules, and were non-pathogenic to the smaller animals. The writers consider that the two forms are modifications of the same organism, and that posterior basic meningitis may be considered as merely a sporadic form of epidemic cerebro-spinal meningitis, both being caused by the organism known as the diplococcus meningitidis intracellularis (Weichselbaum). This organism is seldom present in pure culture in cases of meningitis. In only four of the above nine cases was the diplococcus found alone. In three the bacillus influenza was present, in two cocci, and in one the tubercle bacillus.

**Diphtheria.**—Hewlett and Murray[4] examined the throats of 385 children under the age of 14 years admitted to the Victoria Hospital for diseases other than diphtheria. In 92 or 24 per cent. the pseudo-diphtheria bacillus was found; in 58 or 15 per cent., the Klebs-Löeffler. In children under two years of age they estimate that one out of every five is a possible source of infection; whilst in children over two years of age one out of every eight is infected. In only seven cases was there any clinical evidence of the disease. Three cultures tested by inoculation showed only feeble virulence. The writers give results found by other observers, viz., Löeffler, four infected cases amongst 160 healthy children; Paxke and Beebe 8 cases of virulent bacilli, 24 of non-virulent bacilli, and 27 of pseudo-diphtheria bacilli in 250 children. Rober found diphtheria bacilli in 2·5 per cent. of 600 healthy persons, and in 8 per cent. of 128 persons who had been exposed to infection.

**Bacteriological Examination of the Blood.**—Under the older method of examination, namely, by puncturing the finger and making agar slant cultures from the resulting drop of blood, there was undoubtedly a great risk of contamination from organisms present in the skin. The quantity of blood so taken was extremely minute, so that the chance of detecting the specific organism was small. Some interesting results were, however, obtained by this method. Jakowski[5] examined the blood of nine phthisical patients suffering from hectic and found pyogenic cocci in seven. The organisms found were staphylococcus aureus twice, staphylococcus aureus and streptococcus once, streptococcus twice, staphylococcus aureus and albus twice. Canon,[6] in 14 cases of sepsis, found staphylococcus albus three times, aureus twice, streptococcus seven times, pneumococcus once, and pneumobacillus once. He also found staphylococcus albus in three cases of phlegmon and aureus to two of osteomyelitis, one of which died. The present method of examining the blood consists in distending a vein by tying a ligature around the upper arm, sterilising the skin over the vein, and plunging into it the needle of a sterile syringe and drawing off from 5 to 10 c.c. of blood, which is then spread over agar plates, or mixed with broth and incubated. The results obtained by this method are certainly more reliable than those obtained by the older procedure. Kohn's[7] results are as follow:—Of 54 cases examined 16 gave positive results and the remainder were negative. Of the positive cases two were cases of infective endocarditis. In one staphylo- and streptococci were found, and in the other staphylococci only. Staphylococci were also found in the blood of a case of subacute endocarditis. A bacillus, probably the typhoid bacillus, was found in two cases of enteric fever, and from the blood of a patient suffering from acute pyæmia streptococci were cultivated. In 10 cases of pneumonia the pneumococcus was demonstrated in the general circulation, seven of these died. The series of negative results included subacute endocarditis (2), erysipelas, phlegmon, icterus, hæmoglôbinuria, aphthous stomatitis, enteric fever (6), leucæmia (2), acute rheumatism (2), and 21 cases of pneumonia, three of which died. Blick[8] records a case of enteric fever which ended fatally, and in which during life the bacillus typhosus was demonstrated in the blood on two occasions. Girandeau, in a case of gastric ulcer with septicæmia, found staphylococcus citreus in the blood, the point of infection having been presumably the ulcer. Triboulet and Coyan,[9] examining the blood of a series of cases of acute rheumatism, found a diplococcus present in every instance. Symes[10] found organisms in the blood in five out of 19 cases examined. Four of the cases giving positive results died; they included two of ulcerative endocarditis, one of puerperal sepsis, and one of pneumonia. In the fifth case there was the possibility of accidental contamination. Of the 14 negative cases, two died. Amongst the 14 were cases of puerperal fever, pernicious anæmia, ulcerative endocarditis, cerebro-spinal meningitis and enteric fever. Carr[11] has reported a case of septic infection from a gastric ulcer, the staphylococcus aureus being present in the blood. He also reports two blood infections associated with infective endocarditis, the pneumococcus and the streptococcus being the infective agents. In seven consecutive cases of infective endocarditis, organisms were demonstrated in the blood either during life or immediately after death. A summary of the results of bacteriological examinations of the blood may be found in an article by Ely in the *American Journal of Medical Sciences*, October, 1897.

**Notes.**—Cocaine and atropine solutions[12] such as are used in ophthalmic practice quickly become infected with micro-organisms. They cannot be sterilised by boiling, as this alters their chemical characters and renders them inert. They are best preserved by the addition of 45 per cent. alcohol; atropine or cocaine 3 grams in 20 grams of 45 per cent. alcohol. A new method of staining cilia is described by De Rossi.[13] Agar cultures not more than four days old are used. After carefully diluting very fine films are spread but not fixed. The mordant is a solution of tannic acid 25 grams in aqueous solution of caustic potash (1 per cent.) 100 grams. The stain is Ziehl's original carbol. fuchsin. One drop of the mordant and four of the stain are poured together on the film and left for from 20 to 25 minutes. Wash with distilled water, dry and mount in balsam.

[1] Berl. Klin. Woch, No. 28, 1900. [2] Rep. of Med. Off. of L. Gov. Brd., 1899–1900. [3] Lancet, June 1, 1901, p. 1525. [4] Brit. Med. Jour., June 15, 1901. [5] Central. f. Bakt., Dec. 9, 1893. [6] Deut. Med. Woch, Sept. 26, 1893. [7] Ibid., Feb. 25, 1897. [8] Bull. Johns Hopk. Hosp, June, 1897. [9] Lancet, Jan. 15, 1898. [10] Brit. Med. Jour., Feb. 23, 1901. [11] Edin. Med. Jour., Aug., 1899. [12] Mun. Med. Woch., March 19, 1901. [13] Epit. 36, Brit. Med. Jour. Jan. 12, 1901.

